# Investigating bacterial populations in styrene-degrading biofilters by 16S rDNA tag pyrosequencing

**DOI:** 10.1007/s00253-014-5868-3

**Published:** 2014-06-21

**Authors:** Kevin J. Portune, M. Carmen Pérez, F. Javier Álvarez-Hornos, Carmen Gabaldón

**Affiliations:** Research Group GI2AM, Department of Chemical Engineering, Universitat de València, Av. de la Universidad s/n, 46100 Burjassot, Spain

**Keywords:** Biofiltration, Styrene, Pyrosequencing, FISH

## Abstract

Microbial biofilms are essential components in the elimination of pollutants within biofilters, yet still little is known regarding the complex relationships between microbial community structure and biodegradation function within these engineered ecosystems. To further explore this relationship, 16S rDNA tag pyrosequencing was applied to samples taken at four time points from a styrene-degrading biofilter undergoing variable operating conditions. Changes in microbial structure were observed between different stages of biofilter operation, and the level of styrene concentration was revealed to be a critical factor affecting these changes. Bacterial genera *Azoarcus* and *Pseudomonas* were among the dominant classified genera in the biofilter. Canonical correspondence analysis (CCA) and correlation analysis revealed that the genera *Brevundimonas*, *Hydrogenophaga*, and *Achromobacter* may play important roles in styrene degradation under increasing styrene concentrations. No significant correlations (*P* > 0.05) could be detected between biofilter operational/functional parameters and biodiversity measurements, although biological heterogeneity within biofilms and/or technical variability within pyrosequencing may have considerably affected these results. Percentages of selected bacterial taxonomic groups detected by fluorescence in situ hybridization (FISH) were compared to results from pyrosequencing in order to assess the effectiveness and limitations of each method for identifying each microbial taxon. Comparison of results revealed discrepancies between the two methods in the detected percentages of numerous taxonomic groups. Biases and technical limitations of both FISH and pyrosequencing, such as the binding of FISH probes to non-target microbial groups and lack of classification of sequences for defined taxonomic groups from pyrosequencing, may partially explain some differences between the two methods.

**Electronic supplementary material:**

The online version of this article (doi:10.1007/s00253-014-5868-3) contains supplementary material, which is available to authorized users.

## Introduction

Styrene is a highly volatile organic compound (VOC) that is a commercially important feedstock used in the production of plastics, latex paints and coatings, synthetic rubbers, polyesters, and styrene-alkyd coatings (Miller et al. 1994). Aside from its beneficial uses, short- and long-term exposure to even low styrene concentrations can produce adverse effects such as irritation of the skin, eyes, and respiratory tract; depression of the central nervous system (CNS); headache; fatigue; nausea; and dizziness and can be acutely neurotoxic at high concentrations (World Health Organization Air Quality Guidelines 2000). Although evidence is limited, several occupational studies have shown styrene to be a suspected carcinogen, causing increased risks for lymphohematopoietic cancers and genetic damage to white blood cells and lymphocytes (World Health Organization Air Quality Guidelines 2000; U.S. Department of Health and Human Services 12th Report on Carcinogens 2011). Due to its high vapor pressure (0.67 kPa at 20 °C), proper management and disposal of styrene waste vapor from point sources before its emission to the environment is essential in styrene-utilizing industries. The elimination of styrene waste vapor through biofiltration provides a cost-effective and environmentally friendly alternative to conventional disposal methods (Devinny et al. 1999; Delhomenie and Heitz 2005). The optimization of styrene-degrading biofilters is necessary for maximum styrene elimination and cost-saving measures, thus warranting a comprehensive examination of the microbial communities within biofilters to enhance overall bioreactor performance.

Microbial communities in biofilters make up complex and structured ecosystems that can adapt and change in accordance with variable physicochemical operational conditions. Inoculum sources of microorganisms typically consist of resident microbes found in the organic solid support of the bioreactors, such as peat, compost, woodchips, or from an added source of complex material such as activated sludge (Ralebitso-Senior et al. 2012). Thus, bioreactors start with unique microbial ecosystems that have the potential to respond differently to operational conditions, depending on the structure of the populations. Deciphering the relationship between microbial diversity and environmental parameters is of great importance to better understanding the biological component of biofilters (Malhautier et al. 2005). Many previous studies have attempted to assess this relationship by inferring microbial diversity in bioreactors detected through molecular methods such as cloning (Friedrich et al. 2002), banding patterns from denaturing gradient gel electrophoresis (DGGE) (Li and Moe 2004; Cabrol et al. 2012), fluorescence in situ hybridization (FISH) (Friedrich et al. 2003), single-strand conformation polymorphism (SSCP) analysis (Khammar et al. 2005), (automated) ribosomal intergenic spacer analysis (ARISA) (Borin et al. 2006), and terminal restriction fragment length polymorphism (T-RFLP) analyses (Briones et al. 2007). Although these techniques have provided some insights into characterizing the dominant microbial populations within engineered ecosystems, they provide only limited information on the complete bacterial community structure found within the examined biofilms due to many technological limitations of the measuring methods (Talbot et al. 2008; Ralebitso-Senior et al. 2012).

Most of the previous studies examining the degradation of styrene gas through biofiltration have focused on using either one or a few styrene-degrading bacterial (Okamoto et al. 2003; Iwanade et al. 2005; Jung and Park 2005; Jang et al. 2005, 2006) or fungal strains (Cox et al. 1997; Qi et al. 2002; Rene et al. 2010a, b, 2011) as the inoculum for biofilters. A few studies have identified several bacterial taxonomic groups from styrene-degrading biofilters with an organic packing material using cultivation-based techniques (Arnold et al. 1997) and FISH (Friedrich et al. 1999). However, a complete detailed analysis of complex microbial populations present in styrene-degrading waste gas biofilters has yet to be conducted.

The advent of powerful next-generation DNA sequencing technologies such as pyrosequencing provides the ability to obtain a much more detailed view of microbial consortiums and their structure by examining bacterial sequences at orders of magnitude greater than by using traditional methods such as cloning and Sanger sequencing (Rothberg and Leamon 2008). The application of pyrosequencing to biofiltration studies permits the analysis of changes in microbial structure not only for the dominant groups present in bioreactors but also changes in the entire microbial community as a response to altering operational conditions. Although the use of pyrosequencing is increasingly being applied to diverse ecological studies, this technique has been used in relatively few studies in waste gas biofiltration (Kim et al. 2012, 2013a, b; Moe et al. 2013) with no known studies up to date associated with styrene degradation.

As molecular tools become increasingly applied to study microbial populations in bioreactors, a demand in accuracy and reproducibility of these tools becomes apparent. Known limitations and biases of each of these molecular techniques (Talbot et al. 2008) have the potential to obscure conclusions, yet studies in biofiltration have often only employed one molecular technique to examine microbial communities. Furthermore, PCR-based molecular methods, such as 16S ribosomal RNA (rRNA) amplicon pyrosequencing, are potentially subject to bias associated with DNA extraction and PCR (Polz and Cavanaugh 1998; Feinstein et al. 2009) and variable rRNA operon copy numbers between different phylogenetic species (Fogel et al. 1999). Other molecular techniques that do not rely on a PCR amplification step, such as FISH, can circumvent these PCR-based biases by directly targeting microbial cells within complex samples. Furthermore, FISH can be used for characterizing the spatial arrangement of microbial communities within their natural habitats (Wagner et al. 2003). However, FISH is subject to its own limitations such as a lack of detection of target cells due to low rRNA content or impermeability of bacterial species to FISH probes (Wagner et al. 2003) as well as a lack of complete coverage for broad taxonomic groups from commonly used probes (Loy et al. 2007). These limitations can substantially influence the quantification of targeted taxonomic groups using FISH. Therefore, it is useful to simultaneously apply and compare results from several molecular methods, such as pyrosequencing and FISH, in an effort to more accurately assess and characterize microbial communities within complex environments such as biofilters.

This study was undertaken to apply 16S rDNA tag pyrosequencing to assess the microbial community structure in a laboratory-scale styrene-degrading biofilter undergoing dynamic operational conditions that simulate transient conditions commonly found in industrial production. Results obtained through pyrosequencing were subsequently compared to abundance data of selected microbial taxa identified through fluorescence in situ hybridization (FISH) in another study (Pérez et al. 2014) in order to assess the utility and potential limitations and biases of each method in analyzing microbial populations from biofilter samples.

## Materials and methods

### Biofilter operation conditions and sample collection

A biofilter was constructed with a 14-L methacrylate column (13.6-cm internal diameter, 97-cm total length) packed with fibrous peat (ProEco Ambiente, Spain). Compressed air was pumped through a mass flow controller (Bronkhorst Hi-Tec, The Netherlands) to adjust the gas flow rate and empty bed residence time (EBRT). The air then passed through a humidifier before mixing with styrene supplied through a syringe pump (New Era, infusion/withdraw NE 1000 model, NY, USA). The styrene/air mixture entered the biofilter at the top of the column and exited the cylinder through an outlet gas line. Along the length of the column, five analysis ports were evenly spaced (0, 25, 50, 75, and 97 cm from the styrene inlet port) to facilitate daily measurements of styrene gas concentrations using a total hydrocarbon analyzer (Nira Mercury 901, Spirax Sarco, Spain). The response factor of the total hydrocarbon analyzer was determined by a gas chromatograph (model 7890, Agilent Technologies, Santa Clara, USA) equipped with a 1-ml automated gas valve injection system and a flame ionization detector and a Rtx®-VMS capillary column (30 m × 0.25 mm × 1.4 μm). Helium was used as the carrier gas at a flow rate of 1.3 ml min^−1^. Temperatures of the injector, oven, and detector were 250, 100, and 240 °C, respectively. A 10-cm head space was used for the styrene gas inlet and for water/nutrient feed, while a 10-cm bottom space was used for the treated air outlet and leachate. Fifty milliliters of nutrient solutions buffered at pH 7 (22.4 g of KNO_3_ L^−1^, 2.4 g of KH_2_PO_4_ L^−1^, 0.4 g of K_2_HPO_4_ L^−1^, 0.9 g of MgSO_4_ · 7H_2_O L^−1^, and trace amounts of Ca, Fe, Zn, Co, Mn, Na, Ni, B, I, Se, Cr, Cu, and vitamins) were poured daily into the biofilter at the top of the column in order to support microbial growth. The moisture content of the peat was measured using the dry weight method and was maintained by adding 50 ml of deionized water per day to the top of the biofilter. Leachate was collected at the bottom of the biofilter through a separate outlet line. The pH of the leachate was monitored daily using a pH/Cond 340i multimeter (WTW, Germany).

In order to establish a microbial community that was capable of high removal efficiency, a start-up period was conducted at the beginning of the experiment using styrene inlet loads ranging from 12 to 24 g m^−3^ h^−1^ and EBRTs ranging from 90 to 120 s. Once suitable removal efficiencies were obtained, four different stages of fixed styrene inlet loads and EBRTs were applied (Table 1) to examine the effects of increasing styrene inlet loads at two different EBRTs on biofilter performance. From stages 1 to 2, styrene inlet loads were increased from 22 to 45 g m^−3^ h^−1^, and EBRTs were maintained at 60 s (Table 1). From stages 3 to 4, EBRTs were maintained at 45 s while styrene inlet loads were increased from 22 to 45 g m^−3^ h^−1^ (Table 1). A fifth stage was applied to run the biofilter with minimal maintenance to simulate low industrial emissions during periods of minor or no production. During this stage, the EBRT was kept at 120 s, and the styrene inlet loads were maintained at 8 g m^−3^ h^−1^ (Table 1). Only 100 ml of water and 100 ml of nutrient solution were supplied once per week during this stage.

**Table 1:** Experimental design of biofilter operational parameters

	Styrene inlet load (g m^−3^ h^−1^)	Empty bed residence time (s)	Styrene inlet concentrations (mg Nm^−3^)
Start-up	12–24	90–120	300–790
Stage 1	22	60	335
Stage 2	45	60	750
Stage 3	22	45	275
Stage 4	45	45	560
Stage 5	8	120	267

As part of a separate study that compared the effects of different inoculation procedures on system performance of biofilters (Pérez et al. 2014), the biofilter was inoculated on day 0 with a 1-L culture of the bacterium *Pseudomonas putida* (Trevisan 1889) Migula 1895 strain CECT 324, growing in nutrient broth II medium. Strain CECT 324 was supplied from the Spanish Type Culture Collection (CECT) and was confirmed to degrade styrene after cultivation on minimal salt agar medium (7.0 g of K_2_HPO_4_, 3.0 g of KH_2_PO_4_, 1.0 g of (NH_4_)_2_SO_4_, 2 ml of 10 % (wt/vol) MgSO_4_ · 7H_2_O per liter of QH_2_O, 1.5 % agar) with the addition of 5 μl of styrene as the sole carbon source (data not shown). Prior to inoculation, the culture was aerated for 30 days using non-sterile air continuously fed with styrene at a rate of 0.15 ml h^−1^.

Duplicate biofilm samples were taken during the middle periods of each experimental stage (stages 1, 3, and 4) at 105, 142, and 156 days, respectively, and at the end of stage 5 at 239 days of biofilter operation (a total of eight samples). Samples were taken during stage 2 but were lost for analysis and therefore not used in this study. Biofilm samples were taken from the organic solid support with sterilized tweezers through a port at the bottom of the biofilter (80 cm from the styrene inlet port). Biofilm samples were stored frozen in 2-ml cryovials until nucleic acid extraction.

### Nucleic acid isolation and 16S rDNA 454 pyrosequencing

DNA from each duplicate sample was extracted with a FastDNA Spin Kit for Soil (MP Biomedicals, Illkirch, France) using the manufacturer’s protocol. Prior to pyrosequencing, extracted DNA from each sample was amplified in triplicate reactions via PCR (30-μl final volume), and amplicons were pooled to reduce stochastic variability between reactions. Reactions consisted of final concentrations of 1X PCR buffer, 0.2 mM dNTPs, 0.3 μM forward and reverse primers targeting the V4 hypervariable region of the 16S rRNA gene, and 0.75 units PrimeStar HS DNA polymerase (Takara Bio, Ōtsu, Shiga, Japan). Forward PCR primers consisted of a 454 adapter A sequence (5′-CCATCTCATCCCTGCGTGTCTCCGACTCAG-3′), followed by unique 11 nt sequence barcodes (Table S1 in Electronic supplementary material (ESM)) for each sample, and then the forward primer 563F (5′-AYTGGGYDTAAAGNG-3′) corresponding to *Escherichia coli* (*E. coli*) positions 563–578 (Zhang et al. 2012). Sequence barcodes allowed sample multiplexing to be carried out in a single pyrosequencing run (Binladen et al. 2007). Reverse PCR primers consisted of the 454 adapter B sequences (5′-CCTATCCCCTGTGTGCCTTGGCAGTCTCAG-3′) attached immediately upstream of a mixture of four reverse primers (802R) in equimolar concentrations (5′-TACCRGGGTHTCTAATCC-3′), (5′-TACCAGAGTATCTAATTC-3′), (5′-CTACDSRGGTMTCTAATC-3′), and (5′-TACNVGGGTATCTAATC-3′) corresponding to *E. coli* positions 785–802 (Zhang et al. 2012). PCR cycling conditions consisted of 95 °C for 3 min, followed by 30 cycles of 95 °C for 45 s, 55 °C for 45 s, and 72 °C for 45 s, and a final extension at 72 °C for 10 min. PCR amplicons were purified using a High Pure PCR Product Purification Kit (Roche, Basel, Switzerland), then analyzed with a bioanalyzer (Agilent Technologies). Amplicons were further purified and size selected using Ampure XP beads (Agencourt Bioscience, Beckman Coulter, Brea, CA, USA) before emulsion-based clonal amplification PCR (emPCR). Purified amplicons from all samples were mixed in equal proportions and sequenced on a 454 GS Junior System (Roche). All replicate 1 samples from each of the four time points were run in a single pyrosequencing run (total = 4 samples per run), followed by a separate pyrosequencing run with all replicate 2 samples.

### Pyrosequencing processing and analysis

All sequences of the pyrosequencing runs were deposited in the NCBI Sequence Read Archive (SRA) database (accession no. SRP036184). Samples were analyzed using tools within the software Mothur (version 1.31.2) (Schloss et al. 2009) using a modified version of the protocol (Schloss et al. 2011) found in the Mothur homepage (date accessed June 2013). Pyrosequencing flowgrams (sff files) were trimmed to 340 flows, and sequencing noise was removed using Mothur’s version of PyroNoise (Quince et al. 2011). Sequences were discarded if they were shorter than 150 bp, contained ambiguous bases, or did not contain exact matches to primers and barcodes. Sequences were sorted into respective samples according to each barcoded primer, and then primers and barcodes were removed from each sequence. Sequences were aligned to the SILVA-based bacterial reference alignment (Schloss 2009) and then pre-clustered using Mothur’s version of a pseudo-single linkage algorithm (Huse et al. 2010). Chimeras were removed using Mothur’s version of UCHIME (Edgar et al. 2011). Remaining sequences that originated from mitochondria, chloroplast, or archaea were identified using the classify.seqs command in Mothur with the mothur-formatted version of the Ribosomal Database Project (RDP-II) training set (v.9) (Wang et al. 2007) and then removed from the dataset to produce the final processed sequences. Using Mothur’s dist.seqs and cluster commands, a distance matrix was generated, and sequences were clustered into operational taxonomic units (OTU_0.03_) at a 97 % similarity level using average linkage clustering. Representative sequences from OTUs from each sample were generated using Mothur’s get.oturep command. Taxonomic classification of these sequences were carried out using the RDP Classifier (Wang et al. 2007), and a bootstrap confidence estimate threshold of 50 % was set to assign phylogenetic classification (Claesson et al. 2009; Roeselers et al. 2011). A shared file of OTUs among samples was generated by the make.shared command in Mothur and was used for OTU-based analysis of alpha and beta diversity. This shared file was used to generate rarefaction curves using Mothur’s rarefaction.single command. Diversity indices (Inverse Simpson [1/D] and Shannon diversity index [H′]), richness estimators (Chao1 and the abundance-based coverage estimator [ACE]), observed OTUs, and Good’s coverage for each sample were calculated using the shared file described above with the summary.single command in Mothur. Diversity indices, richness estimators, observed OTUs, and Good’s coverage were calculated by normalizing to the lowest number of sequences for all four sampling time points (i.e. 10,813) and calculating 95 % confidence intervals for the estimators from 1,000 iterations of the subsamples. Evenness (*E*) was calculated based on the Inverse Simpson’s index (*D*) for each replicate sample (Mulder et al. 2004):

$$ E= D/ S=\left(1/{\displaystyle {\sum}_{i=1}^{\mathrm{S}}{P}_i^2}\right)/ S, $$

where *S* represents the number of observed OTUs, and *P*_*i*_ is the proportion of *S* made up of the *i*th species. Evenness (*J*′) was also calculated using Pielou’s evenness index, (*J*′ = *H*′/(ln*S*)), where *H*′ is the Shannon diversity index derived for each sample, and *S* represents the number of observed OTUs. Using the tree.shared command in Mothur, a distance matrix was calculated using the Bray–Curtis dissimilarity and the shared OTU file described above. This distance matrix was then used in hierarchical clustering by the unweighted pair group method with arithmetic mean (UPGMA) algorithm in order to generate a dendrogram to assess similarity between communities of different samples.

### Statistical analysis

Statistical analyses were performed using the software program IBM SPSS Statistics (version 19) (IBM, Chicago, IL, USA) in order to test for significant differences in diversity indices (Inverse Simpson and Shannon’s index) and richness estimators (Chao1 and ACE) between sampling days. Data were initially analyzed for normal distributions using Shapiro–Wilk’s test. Kruskal–Wallis tests were used for comparisons of differences in medians among sampling days. Possible correlations between biofilter operational/functional parameters (average styrene inlet and outlet concentrations, styrene inlet loads, pH of leachate, moisture content, average removal efficiency, styrene elimination capacity) and biodiversity measurements (observed OTUs, Inverse Simpson’s index, Shannon’s index, evenness) at different sampling days were analyzed using Spearman’s rank correlation coefficient (*ρ*). Possible correlations between biofilter operational/functional conditions and percentages of the top 40 most abundant detected genera in replicate samples over the four sampling times were also analyzed using Spearman’s rank correlation coefficient (*ρ*). Average values for biofilter operational and functional parameters were calculated from measurements from the start of each stage up until the sampling time point for microbial analysis for each respective stage as follows: 84–105 days, 138–142 days, 152–156 days, and 162–239 days, corresponding to the sampling time points at 105, 142, 156, and 239 days.

In order to visualize variation in community structure with distance, non-metric multidimensional scaling (NMDS) was conducted in *R*, version 3.03 (R Core Team 2014). Using the shared OTU file described above, a distance matrix was produced by the veg.dist command in the *R* package vegan version 2.0–10 (Oksanen et al. 2013) using the Bray–Curtis dissimilarity. The NMDS was created by the isoMDS function in the *R* package MASS (Venables and Ripley 2002) using two selected dimensions. To investigate potential correlations between biofilter operational parameters and microbial community structure, canonical correspondence analysis (CCA) was carried out using the cca() function in the *R* package vegan. Styrene outlet concentration, moisture content, and EBRT were selected as the environmental variables to test for correlations with community structure. Since styrene inlet concentration, inlet load, and removal efficiency autocorrelated with styrene outlet concentration, these variables were not included in the CCA analysis. In order to standardize different units among environmental variables, average values for styrene outlet concentration, moisture content, and EBRT for each sampling time point and percentages of identified bacterial genera across all samples were first log_10_(*x* + 1)-transformed prior to analysis with CCA. Permutation tests with 999 permutations were performed using the function anova.cca() in the vegan package in order to assess significance of results (Oksanen et al. 2013).

### Fluorescence in situ hybridization (FISH)

FISH was carried out in another study (Pérez et al. 2014) according to protocols described previously (Amann et al. 2001) using samples collected simultaneously as the samples used for pyrosequencing in this study, from the same location (sampling port at 80 cm from the styrene inlet port) of the biofilter. Briefly, 5-ml peat/biomass samples were suspended in 15-ml sterile distilled water and dispersed with an Ultra-Turrax (IKA® T18 basic, Germany). For analyzing Gram-negative bacteria, 0.5-ml aliquots from each sampling time point were fixed with 4 % paraformaldehyde while 0.5-ml aliquots were fixed in ethanol (96 %) for analyzing Gram-positive bacteria. Oligonucleotide probes specific for the phyla *Actinobacteria* (HGC69A used with HGC69A-competitor probes) and *Firmicutes* (equimolar mixture of LGC354A, LGC354B, and LGC354C); the classes *Alphaproteobacteria* (probe, ALF968), *Betaproteobacteria* (BET42a used with BET42a-competitor probes), *Deltaproteobacteria* (equimolar mixture of DELTA495a, DELTA495b, and DELTA495c), and *Gammaproteobacteria* (GAM42a used with GAM42a-competitor probes); the genus *Pseudomonas* (PS56a); and general eubacterial probes (equimolar mixture of EUB338, EUB 338II, and EUB 338III) were used to identify each respective microbial group. Phylum-, class-, and genera-specific probes were labeled with Cy3 fluorescent dyes (Thermo Fisher Scientific, Germany) while the general EUB probes were labeled with Cy5. All sequences and optimal percentages of formamide for each probe are listed in probeBase (Loy et al. 2007). Samples were affixed to glass slides, dehydrated in a series of ethanol washes (50, 80, 98 %), and air dried. Hybridizations were carried out at 46 °C for 2 h, simultaneously using a Cy3-labeled probe and a Cy5-labeled EUB mixture probe to detect all eubacteria. Slides were then washed for 18 min at 48 °C, dried, and mounted.

Fluorescence was detected using a confocal laser scanning microscope (FV 1000, Olympus, Japan). Quantification of fluorescence for each taxonomic-specific probe and general eubacterial probe was carried out according to the methods described in Jubany et al. (2009). Briefly, for each sample, images were manually acquired from 30 randomly chosen microscopic fields. Ten randomly chosen microscopic fields were used for negative controls. An automated method for determining the threshold level was employed using a custom MATLAB program developed in Jubany et al. (2009). Automatic quantification was performed for both the taxonomic-specific and general eubacterial probes, and the upper and lower bounds of error from the image analysis process were calculated according to the methods of Jubany et al. (2009). The percentage of total detected bacteria for each taxonomic group was quantified from the fractions of total fluorescence based on surface estimation obtained by the phyla-, class- or genera-specific probes with the general eubacterial probe mixture.

## Results

### Evaluation of biofilter performance

Full evaluation of the biofilter performance was described elsewhere (Pérez et al. 2014). In this work, calculated average values (mean ± standard deviation) of biofilter performance and actual operational conditions were compiled for the biofilter start-up period and five experimental stages as well as averages for the period of days from the start of each stage up to each respective sampling time point for microbial analysis (Table 2). During the five experimental stages, inlet styrene concentrations ranged from 216 to 797 mg Nm^−3^ and outlet styrene concentrations from 0 to 349 mg Nm^−3^. The biofilter start-up period lasted for 84 days during which an average removal efficiency of 54 ± 24 % was observed (Table 2). In accordance with the selected operational parameters described in Table 1, average styrene inlet loads for stages 1 and 3 (22.0 ± 0.8 and 23.7 ± 1.6 g m^−3^ h^−1^) corresponded to average styrene inlet concentrations of 368 ± 12 and 282 ± 19 mg Nm^−3^, respectively (Table 2). Conversely, average styrene inlet loads during stages 2 and 4 (46.3 ± 1.3 and 45.1 ± 1.5 g m^−3^ h^−1^) corresponded to higher observed average styrene inlet concentrations of 782 ± 20 and 590 ± 26 mg Nm^−3^, respectively (Table 2). Among the five stages, styrene outlet concentrations were highest during stage 2, while concentrations in stages 3 and 5 were considerably lower than in stages 1 and 4. The lowest styrene inlet and outlet concentrations were observed during stage 5, with average styrene inlet loads of 7.8 ± 0.9 g m^−3^ h^−1^ (Table 2).

**Table 2:** Biofilter operational conditions (mean ± S.D.). Samples for microbial analysis were taken on days 105, 142, 156, and 239

Stage (days)	Experimental time periods (days)	Styrene inlet load (g styrene m^−3^ h^−1^)	Styrene inlet concentrations (mg Nm^−3^)	Styrene outlet concentrations (mg Nm^−3^)	Empty bed residence time (s)	Removal efficiency (%)	Styrene elimination capacity (g styrene m^−3^ h^−1^)	pH leachate	Moisture content (%)
Start-up	0–84	16.7 ± 4.1	454 ± 150	230 ± 170	90–120	54 ± 24	8.5 ± 2.7	8.8 ± 0.3	80 ± 2
Stage 1 (84–122)	84–105	22.3 ± 0.9	369 ± 13	165 ± 27	60	55 ± 8	12.3 ± 1.8	8.4 ± 0.3	84 ± 1
	84–122	22.0 ± 0.8	368 ± 12	116 ± 72	60	68 ± 20	15.0 ± 4.2	8.2 ± 0.3	83 ± 2
Stage 2 (122–138)	122–138	46.3 ± 1.3	782 ± 20	252 ± 64	60	68 ± 9	32 ± 4.2	8.0 ± 0.2	81 ± 1
Stage 3 (138–152)	138–142	24.5 ± 1.1	291 ± 13	19 ± 3	45	93 ± 1	22.9 ± 1.3	7.8 ± 0.1	81 ± 1
	138–152	23.7 ± 1.6	282 ± 19	14 ± 11	45	95 ± 3	22.6 ± 1.3	8.1 ± 0.0	81 ± 1
Stage 4 (152–162)	152–156	45.0 ± 2.1	603 ± 27	100 ± 15	45	83 ± 2	37.5 ± 2.1	8.1 ± 0.2	57 ± 1
	152–162	45.1 ± 1.5	590 ± 26	153 ± 68	45	74 ± 12	33.3 ± 5.6	7.1 ± 0.5	54 ± 5
Stage 5 (162–239)	162–239	7.8 ± 0.9	263 ± 31	8 ± 4	120	97 ± 1	7.7 ± 0.9	7.9 ± 0.1	67 ± 23

Instantaneous changes in the removal efficiency were observed to correspond to manual changes in the styrene inlet loads, as increases in inlet loads during the transition from stages 1 to 2 and stages 3 to 4 caused sharp reductions in the removal efficiency (full data in Pérez et al. (2014)), while manual decreases in inlet loads from stages 2 to 3 and stages 4 to 5 caused sudden increases in removal efficiency. The average removal efficiencies during stages 3 and 5 (95 ± 3 and 97 ± 1 %, respectively) were the highest among all stages tested (Table 2). Low average removal efficiencies (55 ± 8 %) were also observed in stage 1 during days 84–105 due to a high pressure drop caused by over-compaction of the peat solid support in the biofilter (full data in Pérez et al. (2014)). On day 110, the compaction in the peat solid support was relieved, and the styrene removal efficiency subsequently increased to 95 %, with an average of 68 ± 20 % for days 84–122 (Table 2). The packing material was monitored without styrene feeding, and analysis of total hydrocarbon revealed no detectable signal, thus suggesting no passive emissions from the organic packing material itself.

### Bacterial community analysis by pyrosequencing

The pyrosequencing runs yielded between 14,443 and 31,167 raw reads for replicates from the four sampling time points (Table 3). After sequence processing (i.e., removing chimeras and sequences that did not pass the quality control criteria), these sequences were reduced between 10,813 and 24,018 sequences (Table 3). Using the normalized sequences (10,813), the largest number of observed OTUs was detected in replicate samples 1 and 2 at 142 days (572 and 601 OTUs, respectively) (Table 3). Rarefaction curves for each time point demonstrated that no sample reached an asymptotic level, suggesting that the sequencing depths of up to 24,018 sequences were insufficient in covering the complete bacterial diversity (Fig. S1 in ESM).

**Table 3:** Summary of raw and processed sequences from 454 pyrosequencing, including diversity indices and richness estimators from replicate samples (replicate 1 = R1, replicate 2 = R2) for each of the four sampling time points

Sample	Raw reads	Sequences post-processing	Normalized sequences	OTUs^a^	Inverse Simpson (*1/D*)	Shannon diversity (*H*′)	Chao1 richness estimation	ACE richness estimation	Good’s coverage (%)	Evenness (*E*)^c^	Evenness (*J′*)^d^
105 days R1	17,630	12,816	10,813	492	21.2 (20.3–22.1)^b^	4.12 (4.08–4.15)^b^	784 (691–921)^b^	925 (847–1,022)^b^	98.2	0.0430	0.664
105 days R2	19,466	15,793	10,813	431	18.6 (17.7–19.6)^b^	4.11 (4.07–4.14)^b^	619 (552–722)^b^	702 (646–774)^b^	98.7	0.0432	0.677
142 days R1	14,443	10,813	10,813	572	40.5 (38.9–42.2)^b^	4.59 (4.56–4.63)^b^	787 (722–881)^b^	825 (762–910)^b^	98.1	0.0708	0.724
142 days R2	22,390	18,876	10,813	601	49.6 (47.7–51.7)^b^	4.75 (4.72–4.78)^b^	865 (786–978)^b^	986 (914–1,074)^b^	98.0	0.0825	0.743
156 days R1	31,167	24,018	10,813	455	19.7 (19.1–20.5)^b^	3.92 (3.88–3.95)^b^	769 (668–919)^b^	943 (855–1,049)^b^	98.2	0.0434	0.640
156 days R2	25,691	21,274	10,813	444	18.5 (17.7–19.3)^b^	3.95 (3.91–3.98)^b^	638 (574–735)^b^	669 (612–746)^b^	98.5	0.0416	0.648
239 days R1	17,579	14,131	10,813	425	9.9 (9.5–10.2)^b^	3.44 (3.40–3.48)^b^	692 (605–822)^b^	882 (799–984)^b^	98.3	0.0232	0.568
239 days R2	21,179	17,887	10,813	542	34.8 (33.5–36.2)^b^	4.48 (4.45–4.51)^b^	776 (702–885)^b^	764 (708–841)^b^	98.3	0.0641	0.712

^a^Operational taxonomic units observed after normalization

^b^95 % confidence intervals of respective estimators shown in parentheses

^c^Evenness based on Inverse Simpson index

^d^Evenness based on Shannon index

Values of the bacterial diversity indices Inverse Simpson (*1/D*) and Shannon diversity (*H*′) were substantially higher at 142 days compared to the other time points; however, no significant differences (*P* > 0.05) were observed among time points (Table 3). No significant differences (*P* > 0.05) were also observed in Chao1 and ACE richness estimates among time points (Table 3). The highest values of evenness (*E*) based on the Inverse Simpson’s index (*1/D*) and Pielou’s evenness index (*J*′) were detected at 142 days (Table 3). Using Spearman’s rank correlation coefficient, no significant correlations (*P* > 0.05) were detected between biofilter operational/functional parameters (average styrene inlet and outlet concentrations, styrene inlet loads, pH of leachate, moisture content, average removal efficiency, styrene elimination capacity) and biodiversity measurements (observed OTUs, Inverse Simpson’s index, Shannon’s index, evenness). Good’s coverage values ranged between 98.0 and 98.7 % for all the four time points (Table 3).

**Fig. 1:**
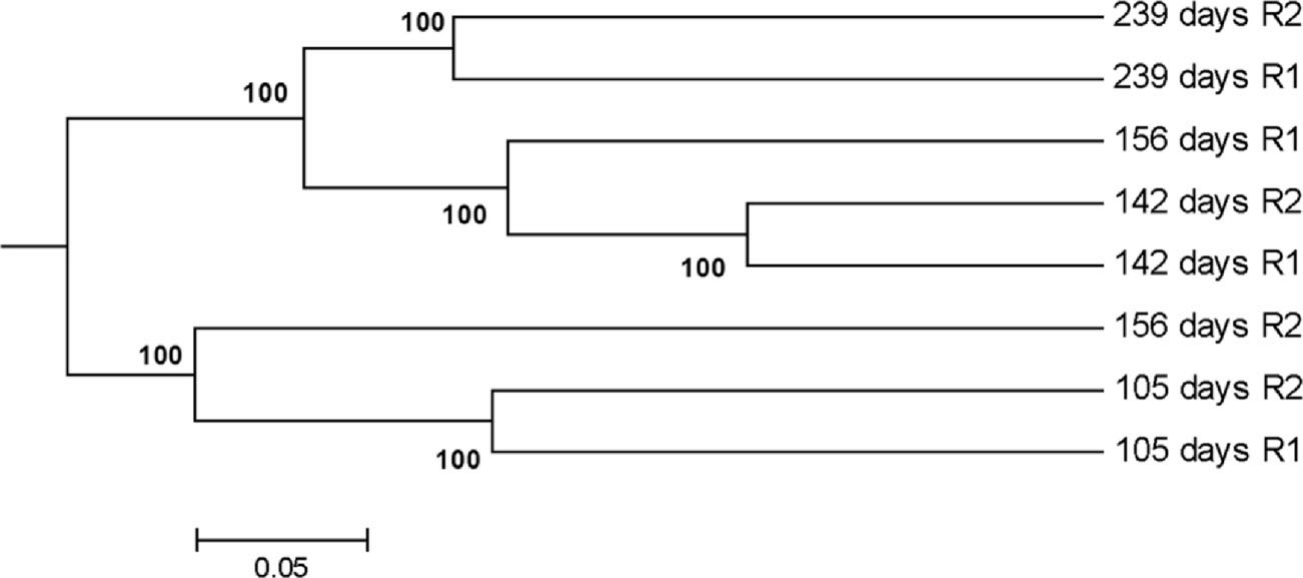
Dendrogram showing the similarity of microbial communities from replicate samples for time points on days 105, 142, 156, and 239 based on Bray–Curtis dissimilarity. Bootstrap analysis of 1,000 iterations was conducted, and bootstrap percentage values are shown on *nodes*

Dendrograms generated from the Bray–Curtis dissimilarity revealed that replicate samples from the same time points clustered together independently of replicates from other time points except replicates from 156 days (Fig. 1). Replicate 1 from 156 days clustered with samples from days 142 and 239, while replicate 2 from 156 days shared more similarities with samples from 105 days (Fig. 1). Analysis of NMDS with pre-selected two dimensions resulted in a minimum stress level of 0.0084. Visualization revealed similar results as the dendrogram, as replicate samples clustered closely together from days 105, 142, and 239, respectively, while replicate 1 from day 156 was spatially closer to replicate samples from days 142 and 239 than to replicate 2 from day 156 (Fig. S2). Replicate samples from days 105 and 239 were highly separated along the first ordination axis (Fig. S2).

### Bacterial taxonomy identified by pyrosequencing

**Fig. 2:**
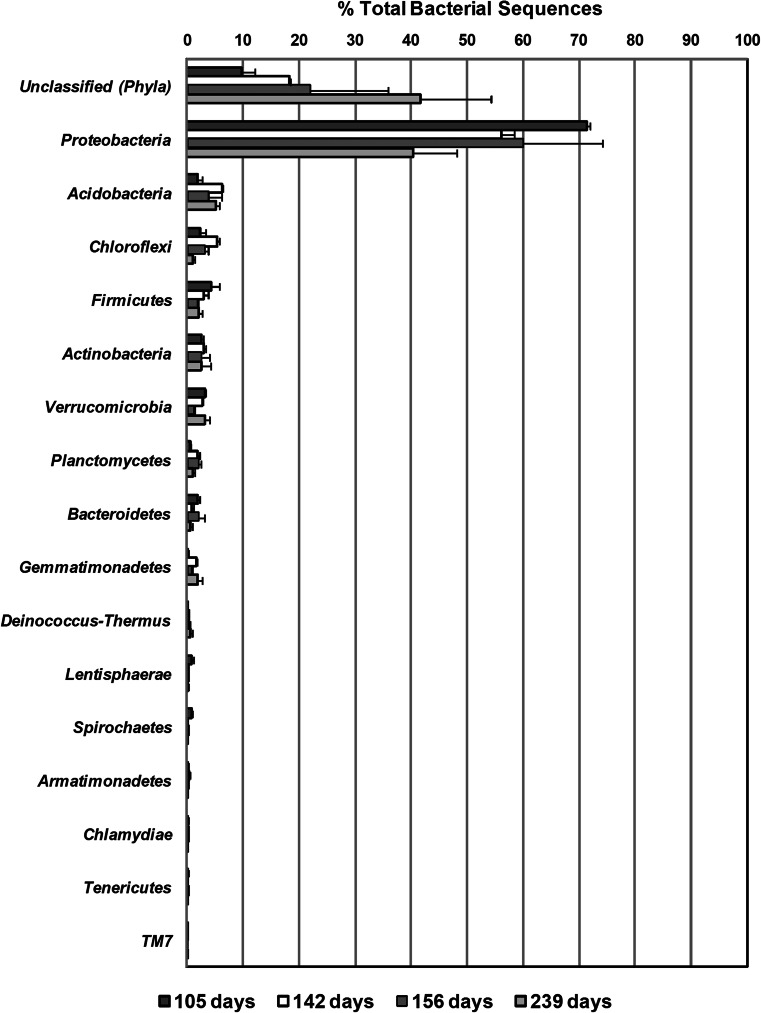
Percentage of total bacterial sequences (mean of replicates) assigned to bacterial phyla in the four sampling time points. *Error bars* represent the lower and upper values of replicates for each sampling time. Sequences labeled as unclassified (*Phyla*) could not be assigned to any classified phylum using the Ribosomal Database Project Classifier at the selected bootstrap confidence estimate threshold of 50 %

Bacteria from 16 different phyla were represented in the biofilter over the four sampling time points, with the majority of total bacterial sequences classified to the phylum *Proteobacteria* (Fig. 2). However, as the biofilter operation proceeded, the average percentage of total bacterial sequences from this group decreased from 71.5 % on day 105 to 41.6 % on day 239 (Fig. 2). Percentages of total bacterial sequences assigned to other phyla were less than 7 % of the total sequences for each respective time point (Fig. 2). The average percentage of sequences that were unable to be assigned to any known phylum at the selected bootstrap confidence estimate threshold level of 50 % rose from 9.8 % on day 105 to 41.6 % on day 239. Further analysis revealed that a large majority of these unclassified sequences (corresponding to an average of 2.7 to 36.6 % of total sequences) displayed sequence similarity to the phylum *Chloroflexi* at bootstrap confidence estimates between 7 and 49 % using the RDP Classifier (data not shown). These sequences were further analyzed using NCBI’s BLASTN tool against the non-redundant nucleotide database (Altschul et al. 1990) and were found to match with up to 98 % sequence identity to uncultured *Chloroflexi* bacterial clones.

**Fig. 3:**
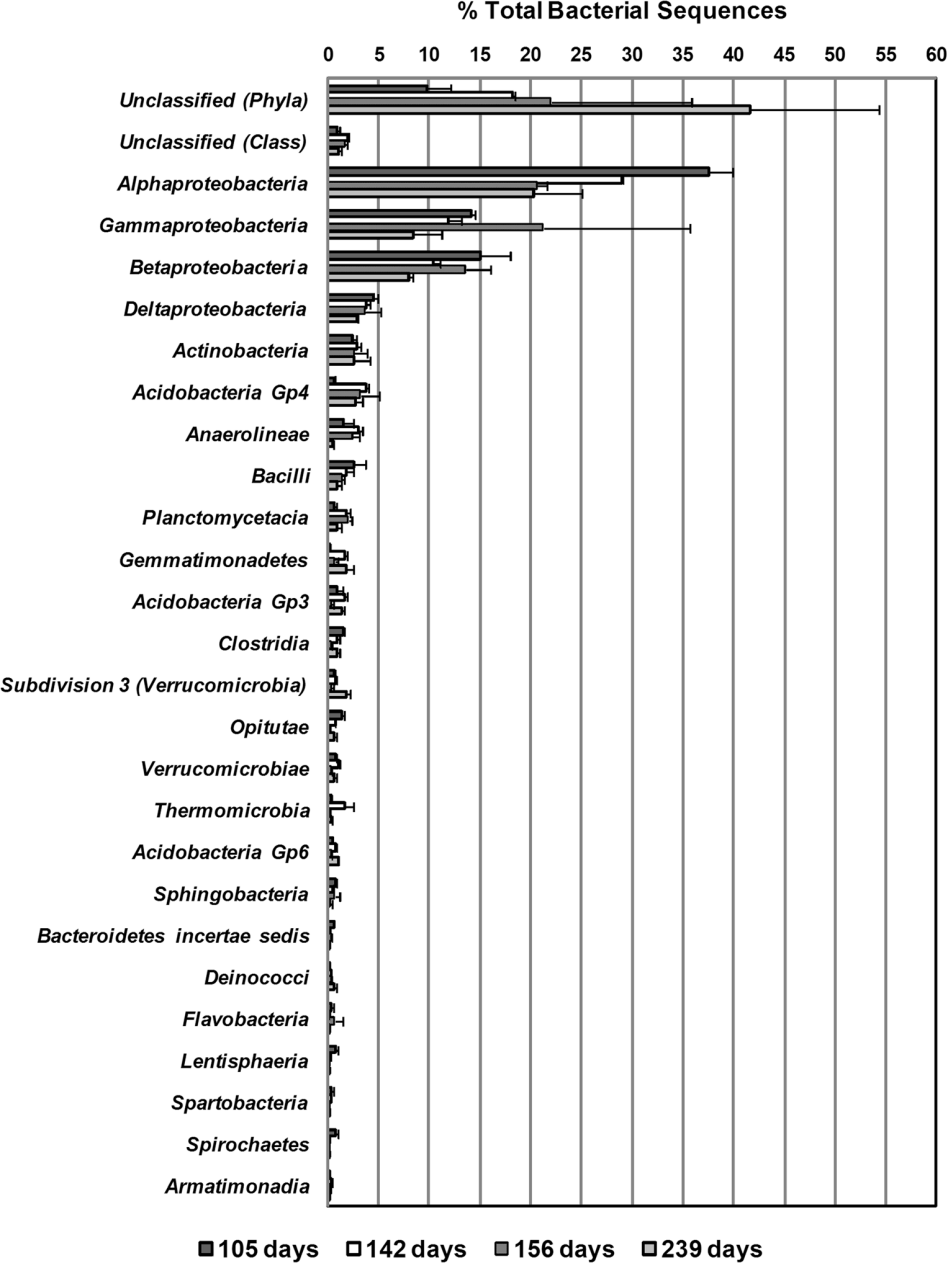
Percentage of total bacterial sequences (mean of replicates) from the top 25 abundant detected classes in the four sampling time points. *Error bars* represent the lower and upper values of replicates for each sampling time. Sequences labeled as unclassified (*Class*) could not be assigned to any particular class using the Ribosomal Database Project Classifier at the selected bootstrap confidence estimate threshold of 50 %

**Fig. 4:**
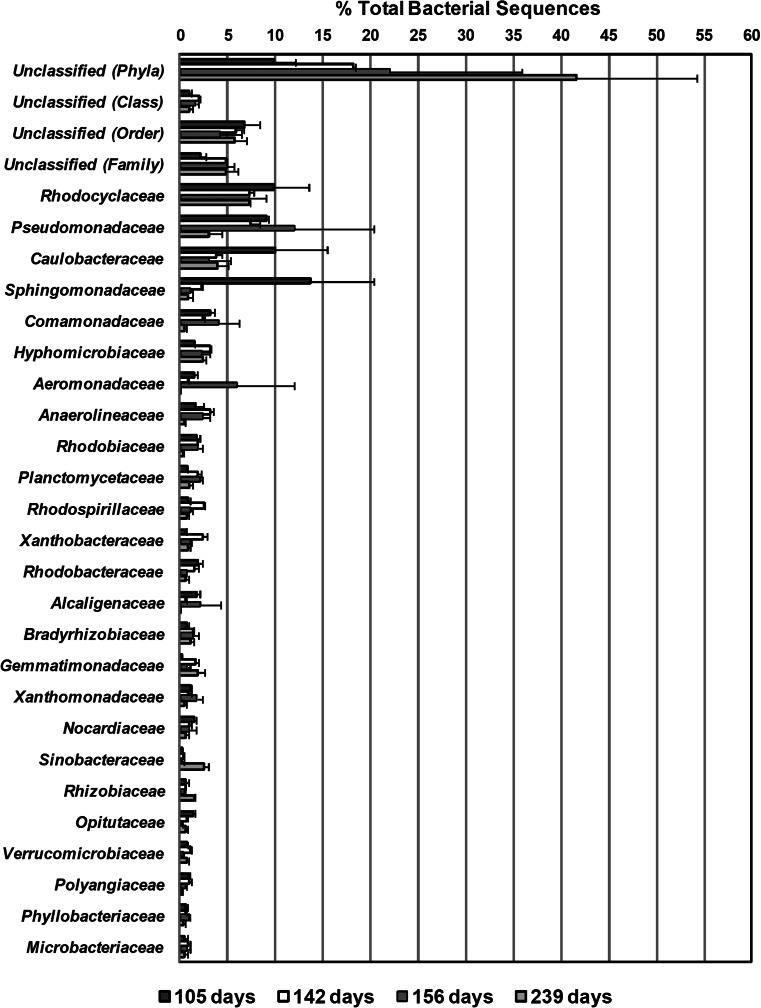
Percentage of total bacterial sequences (mean of replicates) from the top 25 abundant detected families in the four sampling time points. *Error bars* represent the lower and upper values of replicates for each sampling time. Sequences labeled as unclassified (*Family*) could not be assigned to any particular family using the Ribosomal Database Project Classifier at the selected bootstrap confidence estimate threshold of 50 %

Among sequences that could be classified at the class level, *Alphaproteobacteria* was detected as the dominant class of bacteria in all time points excluding 156 days, with average percentages ranging from 20.4 to 37.5 % (Fig. 3). *Gammaproteobacteria* and *Betaproteobacteria* were the next dominant classes (8.5 to 21.2 % and 8.0 to 15.1 %, respectively), followed by *Deltaproteobacteria* (2.9 to 4.5 %) (Fig. 3). The remaining identified bacterial classes were detected in percentages of <6.0 % of total bacterial sequences (Fig. 3). Among the 125 bacterial families represented in at least one of the four sampling time points, *Rhodocyclaceae*, *Pseudomonadaceae*, *Caulobacteraceae*, and *Sphingomonadaceae* were among the dominant bacterial families detected (Fig. 4). The remaining identified bacterial families were detected in percentages of <4.0 % of total bacterial sequences over the sampling period except *Aeromonadaceae* which was detected at averages of 6 % during day 156 (Fig. 4).

At the genus level, 261 genera were classified in at least one of the four sampling time points. *Azoarcus*, *Pseudomonas*, and *Brevundimonas* were among the highest identified bacterial genera over the four time points tested (Fig. S3). Reductions in average percentages of total sequences assigned to *Pseudomonas* and *Brevundimonas* were observed during days 142 and 239, with average percentages dropping to 0.10 and 0.52 %, respectively, for *Pseudomonas* and *Brevundimonas* during the last sampling time point (Fig. S3). Numerous bacterial genera were detected in low abundances in all four time points, ranging between <1 and 5 % of the total bacterial sequences, among them including the known styrene-degrading bacterial genera *Rhodococcus*, *Xanthobacter*, and *Sphingomonas* (Fig. S3). Averages of 8.9 to 23.5 % of total sequences could not be further classified at the genera level at a 50 % cutoff threshold (Fig. S3). Among these sequences, a large majority could be classified at least to the bacterial families *Pseudomonadaceae* (averages from 2.9 to 5.8 % of total sequences per sampling day) and *Sphingomonadaceae* (averages from 0.2 to 12.7 % per sampling day) (Table S2). When these sequences were analyzed using NCBI’s BLASTN tool against the non-redundant nucleotide database, they were found to match with up to 98 % sequence identity to various *Pseudomonas* spp. and *Sphingomonas* spp., respectively (data not shown).

### Relationship between biofilter functional/operational conditions and taxonomy

**Fig. 5:**
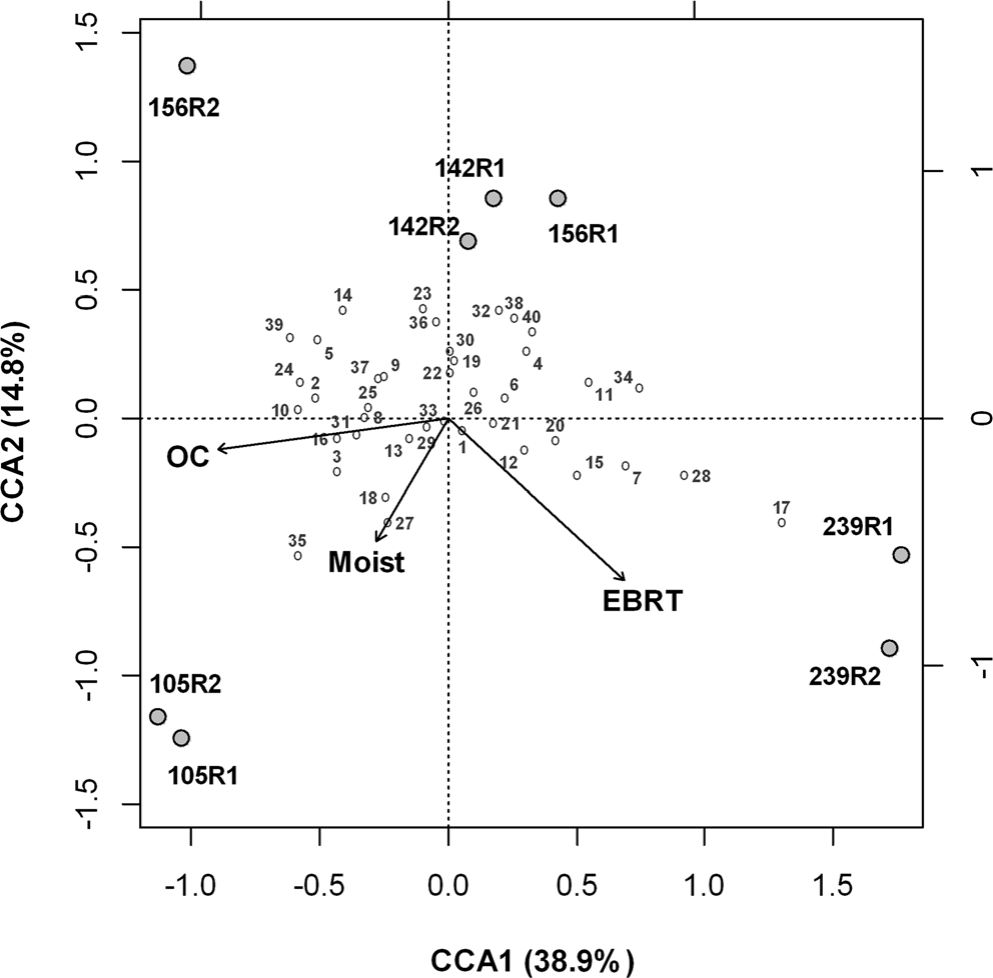
Canonical correspondence analysis (CCA) of bacterial community structure with biofilter operational conditions. *Arrows* represent the variables styrene outlet concentration (OC), moisture content (moist), and empty bed residence time (EBRT) and indicate the direction and magnitude of the variables associated with bacterial community structure. Replicate samples (R1 and R2) over the four sampling time points on days 105, 142, 156, and 239 are represented by *gray circles. Open circles with numbers* represent the top 40 most abundant genera detected over the four sampling time points found in Fig. S3. Percentage variance accounted by each axis is shown in parentheses

CCA produced three constrained axes which accounted for 65.8 % of the explained variance, and the first two axes accounted for 53.7 % of the explained variance. CCA revealed that replicate samples from each sampling time point had similar compositions to each other with the exception of replicates from 156 days which were distinctly separated from each other (Fig. 5). The global permutation test for all constraints together showed the relationship between the genera and environmental variables to be significant (*P* < 0.01 based on 999 permutations). Permutation tests for individual environmental variables revealed styrene outlet concentration and EBRT to be significant (*P* < 0.05) while moisture content was not found to be significant (*P* = 0.170). Styrene outlet concentration was highly correlated with CCA1. Styrene outlet concentrations and EBRT exhibited a greater importance (longer arrows in CCA plot) to the ordination than moisture content (Fig. 5). Numerous bacterial genera were found in conditions of increasing styrene outlet concentrations, among these including *Brevundimonas* (3; the number assigned in the CCA plot corresponding to Fig. S3), *Hydrogenophaga* (8), *Achromobacter* (10), and the known styrene-degrading genus *Pseudomonas* (2) (Fig. 5).

Correlation analysis of biofilter functional/operational parameters with percentages of the top 40 most abundant genera detected in replicate samples over the four sampling times revealed significant correlations (*P* < 0.05) for numerous taxonomic groups (Table S3). *Brevundimonas*, *Hydrogenophaga*, and *Achromobacter* were among the top 10 abundant bacterial genera to have significant (*P* < 0.05) positive correlations with styrene outlet concentrations.

### Comparison of pyrosequencing vs. FISH for bacterial phylogenetic analysis

**Fig. 6:**
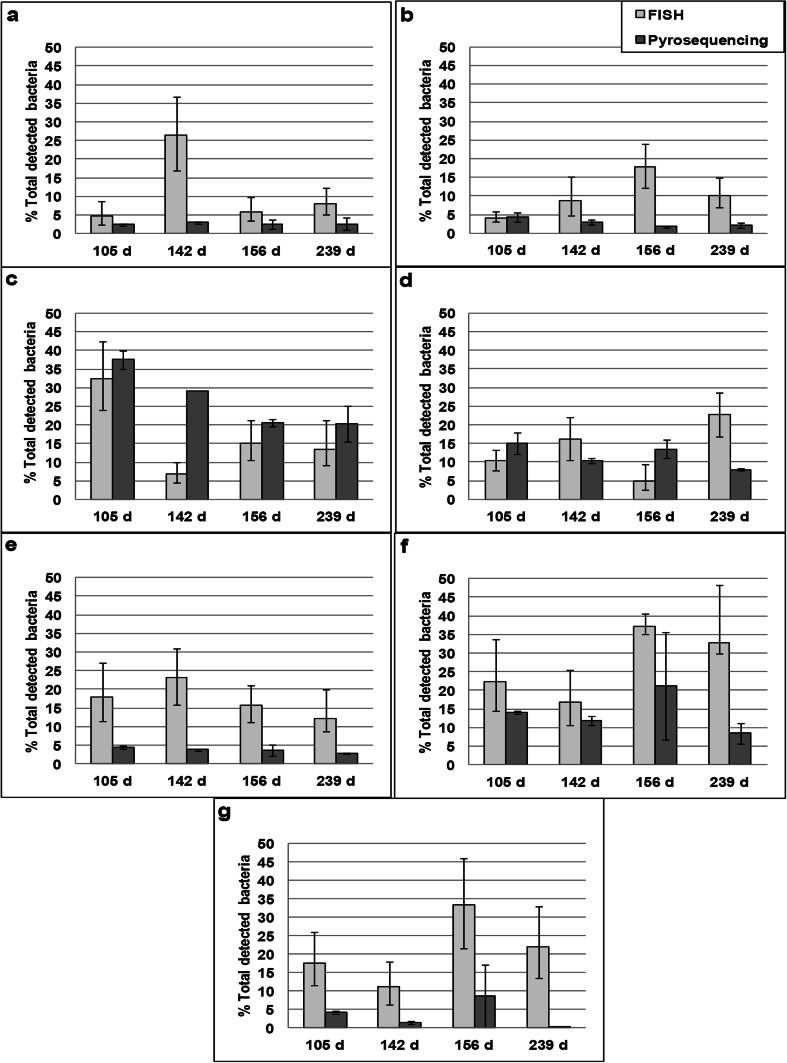
Percentages of total bacteria detected by FISH and pyrosequencing for the taxonomic groups **a** *Actinobacteria*, **b** *Firmicutes*, **c** *Alphaproteobacteria*, **d** *Betaproteobacteria*, **e** *Deltaproteobacteria*, **f** *Gammaproteobacteria*, and **g** *Pseudomonas. Percentages* for pyrosequencing represent the fractions of total sequence tags that could be assigned to a specific taxonomic classification. *Error bars* represent the lower and upper values of individual replicates for each sampling time. *Percentages* for FISH represent the fractions of total fluorescence obtained by general eubacterial probes combined with specific phyla, class, or genera probes. *Error bars* in the FISH data represent the upper and lower bounds of error calculated from the image analysis process. All FISH data originally comes from Pérez et al. (2014)

Comparisons of pyrosequencing and FISH showed different resulting trends between the two methods in detected relative abundances of several taxonomic groups among sampling time points (Fig. 6). For example, a large increase in *Actinobacteria* was observed at day 142 compared to other time points as detected by FISH, whereas no differences in percentages between time points were detected by pyrosequencing (Fig. 6a). *Pseudomonas* spp. were detected by pyrosequencing in very low abundances (<0.2 % total detected bacteria) at day 239, whereas a large percentage of *Pseudomonas* (13–33 %) was detected by FISH at this time point (Fig. 6g). Higher percentages of total detected bacteria assigned to the phylum *Firmicutes*, the class *Deltaproteobacteria*, and the genus *Pseudomonas* were detected by FISH in multiple sampling time points compared to pyrosequencing (Fig. 6b, e, g). In the majority of selected taxonomic groups detected by FISH, high variability was observed in the upper and lower bounds of error calculated from the image analysis process (Fig. 6). In contrast, the variability in detected taxonomic groups between replicate samples by pyrosequencing was much lower than by using FISH, with the exception of day 156 in which high variability between replicates detected by pyrosequencing was observed for the taxonomic groups *Pseudomonas* and *Gammaproteobacteria* (Fig. 6f, g). Some similarities between the two methods were also observed, as pyrosequencing and FISH both identified *Proteobacteria* as the dominant bacterial phylum, with an average of 41.6 to 71.5 % identified by pyrosequencing and 63 to 83 % identified by FISH (Figs. 2 and 6).

## Discussion

Understanding the complex relationships between bacterial community structure and biofilter operational/functional parameters has remained a longstanding challenge in the biodegradation field. The implementation of pyrosequencing in this study permitted an in-depth characterization of the dynamic community structure within a styrene-degrading biofilter, thus enabling a more detailed examination of these relationships. In this study, clustering analysis and NMDS revealed notable changes in community structure among time points, especially between the first and last sampling points during stages 1 and 5, respectively. These observed differences can be attributable to biofilter operational parameters during these stages, as bacteria were exposed to higher styrene inlet and outlet concentrations as well as lower EBRTs during stage 1 compared to stage 5. Similar clustering patterns were also observed in the CCA, as replicate samples from days 105 and 239 were highly separated along the first axis (CCA1) with styrene outlet concentrations highly correlated to the axis, indicating that different ranges of styrene concentrations played a critical factor in the observed differences among these communities.

Bacterial diversity from a temporal point of view has usually been found to be stable over time in waste gas bioreactors and wastewater treatment systems under both constant or disturbed operating conditions (Cabrol and Malhautier 2011 and references therein). However, in systems that degrade recalcitrant compounds such as VOCs, microbial diversity has been observed to both increase (Borin et al. 2006) as well as decrease (Bayle et al. 2009) under selective conditions of increasing pollutant concentrations. Compared to other sampling time points in this study, diversity index values were considerably higher in replicate samples at 142 days, under conditions of relatively low styrene inlet loads and outlet concentrations compared to samples from 105 to 156 days (Table 2). Still, no significant differences (*P* > 0.05) in bacterial diversity indices (Inverse Simpson (*1/D*) and Shannon diversity (*H*′)) were observed between sampling time points. High variation in diversity index values between replicate samples (particularly in replicates from day 239), however, may have considerably influenced the inability to detect significant differences between time points in this study. It is unclear whether biological heterogeneity within biofilms or technical variability within pyrosequencing is the major source of this variability.

Previous studies have shown an inconclusive effect of microbial diversity on bioreactor purification performance, as high performance has been reported in biofilters displaying both high (Ding et al. 2008) and low (Steele et al. 2005) microbial diversities. In addition, either weak or no significant correlations between microbial and waste gas parameters have been reported in studies in industrial biofilters (Friedrich et al. 2003). In this study, no significant correlations were detected between bacterial diversity indices and operational/functional parameters. Although the high variation in diversity index values between replicate samples in this study may have affected the ability to detect potential correlations, other community factors may actually be more important in impacting biofilter function. Numerous studies show that ecosystem function may be more influenced by components of diversity such as species composition with particular traits, positive species interactions, and functional redundancy rather than by microbial diversity itself (Cabrol and Malhautier 2011 and references therein). Furthermore, the large changes in biofilter performance (i.e., immediate increase/decrease in removal efficiency) observed when operational parameters were changed for each respective stage (see Pérez et al. 2014) can be attributed more to the changes in styrene inlet loads and pressure drop rather than sudden changes in microbial populations.

Bacteria from the phylum *Proteobacteria* have previously been identified by DGGE as the dominant phylum in biotrickling filters degrading mixtures of VOCs including styrene (Li et al. 2012; Lebrero et al. 2012). Using domain-, class-, and subclass-specific FISH probes, Friedrich et al. (1999) showed that bacteria from *Actinobacteria* and *Alphaproteobacteria* were detected as the dominant bacterial groups in biofilters degrading styrene. Similar results from pyrosequencing and FISH in this study demonstrated that *Proteobacteria* dominated the biofilter, while *Alphaproteobacteria* were among the dominant classes detected by pyrosequencing. Deeper examination of lower taxonomic levels by pyrosequencing surprisingly revealed the family *Rhodocyclaceae* to be among the dominant families detected in the biofilter during the studied period, with *Azoarcus* as the dominant classified genus. *Azoarcus* is capable of both aerobic and anaerobic growth on a large number of different aromatic compounds including phenylacetate, a well-known intermediate product in the degradation of styrene (Anders et al. 1995), and appears to play an important role in styrene degradation in the biofilter in this study.

Interestingly, CCA and correlation analysis revealed that among the top 10 most abundant classified bacterial genera, *Brevundimonas*, *Hydrogenophaga*, and *Achromobacter* were positively associated with increasing styrene outlet concentrations, suggesting that these genera may play important roles in styrene degradation under increasing concentrations. Genes involved in the styrene-degradation pathways from *Brevundimonas* and *Achromobacter* have been identified in the Kyoto Encyclopedia of Genes and Genomes (KEGG) database (Kanehisa and Goto 2000), while whole genome shotgun sequencing has revealed that a strain of *Hydrogenophaga* sp. contain the gene styA (Szabo et al. unpublished) for styrene monooxygenase, a key component in the upper pathway of styrene catabolism.

High bacterial diversity observed in biofiltration studies may be explained by large numbers of saprophytic microorganisms dominating the biofilter and consuming cellular products, extrapolymeric substances, or the packing material (reviewed in Cabrol and Malhautier 2011). In this study, the large increases in the percentages of unknown bacteria with 16S rRNA sequence similarity to uncultured *Chloroflexi* bacteria indicates that these bacteria were able to utilize favorable growth conditions within the biofilter to proliferate over time. Furthermore, since the highest percentages of these sequences were detected in the last time point at 239 days, when styrene outlet concentrations were very low (<8 mg Nm^−3^), this suggests that that these bacteria were not using styrene as their primary nutritional carbon source. Some members of the *Chloroflexi* phylum have played key roles in submerged membrane bioreactors treating municipal wastewater by eliminating soluble microbial products and cell material largely produced by cellular decay and lysis (Miura et al. 2007). Members of the phylum *Chloroflexi* have also been detected as dominant bacterial groups in biotrickling filters degrading mixtures of volatile organic compounds including methyl mercaptan, toluene, alpha-pinene, and hexane (Lebrero et al. 2012).

Technical reproducibility is an essential factor in the interpretability of results obtained by molecular techniques. Due to the recent implementation of pyrosequencing in microbial ecology studies, few investigations have assessed potential differences in taxonomic assemblages within complex environmental samples such as waste gas biofilters using both pyrosequencing and FISH. In general, the selected taxonomic groups in this study detected by FISH displayed considerably higher variation in percentages of total detected bacteria among sampling time points compared to pyrosequencing. Well-known technical issues associated with detection and quantification of target cells in complex samples using FISH, such as cell aggregation found in biofilms (Wagner et al. 2003) or non-homogenous dispersions of target cells within samples, may be the source of this observed high variation. As DNA extraction is directly carried out on all members within a sample, cell aggregation is not as severe a problem for techniques such as pyrosequencing.

Both agreement (Zheng et al. 2013) and disagreement (Dawson et al. 2012) among relative abundances of several selected taxonomic groups detected by pyrosequencing and FISH have been observed when both techniques were employed in the same studies. For several taxonomic groups in this study, such as *Firmicutes*, *Deltaproteobacteria*, and *Pseudomonas*, larger percentages of total detected bacteria were found using FISH compared to pyrosequencing in most time points. These observed differences may partially be explained by the binding of FISH probes to non-target microbial groups within complex environmental samples. Gougoulias and Shaw (2012) demonstrated that up to 30 % of bacterial cells targeted by the *Pseudomonas*-specific FISH probe PSE1284 were affiliated with the *Burkholderia* spp. The FISH probe DELTA495a used in this study, which targets *Deltaproteobacteria*, is known to target several bacterial taxa outside of its intended target group, including members of the phylum *Gemmatimonadetes* (Loy et al. 2007). Using the software TestProbe within the SILVA comprehensive ribosomal RNA database (Quast et al. 2013), in silico analysis revealed that DELTA495a targets 95 % of the deposited *Gemmatimonas* (phylum *Gemmatimonadetes*) sequences in the SILVA database (data not shown). As *Gemmatimonas* was detected by pyrosequencing in samples from all four time points tested, its presence may have caused an over-estimation of observed *Deltaproteobacteria* by FISH in this study.

A lack of classification of all sequences to the genera level using pyrosequencing may also contribute to some of the observed differences in total detected bacteria between pyrosequencing and FISH. For example, the unclassified sequences within the family *Pseudomonadaceae* (2.9 to 5.8 % of total bacterial sequences) (Table S2) showed high sequence identity (98 %) to various *Pseudomonas* spp. when analyzed using NCBI’s BLASTN. The lack of assignment of these sequences to the *Pseudomonas* genera (Table S2) may partially explain some of the lower percentages of *Pseudomonas* detected by pyrosequencing compared to FISH among multiple sampling time points.

To conclude, microbial communities within biofilters exposed to dynamic operational conditions undergo considerable changes in structure in response to these different conditions. Although significant correlations can be identified between certain bacterial genera and biofilter operating parameters, a definitive relationship between biofilter operational/functional parameters and biodiversity is still unclear. Comparisons of results obtained by pyrosequencing with FISH enabled the identification of inconsistencies between the two methods in detecting certain bacterial taxonomic groups, thus suggesting that the use of only one molecular method to analyze microbial systems in bioreactors has the potential to yield erroneous conclusions.

## Electronic supplementary material

Below is the link to the electronic supplementary material.

ESM 1 (PDF 399 kb)
