# Supplementary material for: Investigating bacterial populations in styrene-degrading biofilters by 16S rDNA tag pyrosequencing
Source: Appl Microbiol Biotechnol. 2014 Jun 21;99(1):3–18. doi: 10.1007/s00253-014-5868-3 (PMC4286631; doi:10.1007/s00253-014-5868-3)
Supplement: Supplementary file 1 — (PDF 399 kb) [file 253_2014_5868_MOESM1_ESM.pdf]

Supplementary Information

Title: Investigating bacterial populations in styrene-degrading biofilters by 16S rDNA tag pyrosequencing

Journal: Applied Microbiology and Biotechnology

Authors: Kevin J. Portune*, M. Carmen Pérez, F. Javier Álvarez-Hornos, and Carmen Gabaldón

Affiliation: Research Group GI^{2}AM, Department of Chemical Engineering, Universitat de València

***Corresponding author:** Kevin Portune
Address: Department of Chemical Engineering, Universitat de València, Av. de la Universitat s/n, 46100, Burjassot, Spain.
Email: kevin.portune@uv.es, Phone: +34 963-543-451; fax: +34963544898

Table S1. Eleven nucleotide sequence barcodes used in PCR for pyrosequencing. Replicate 1 (R1) and replicate 2 (R2) samples were used in separate pyrosequencing runs.

Sample (days of biofilter operation)	Barcode Sequence
105 R1	ACGTACACACT
105 R2	ACGTACACACT
142 R1	ACGTACTGTGT
142 R2	ACGTACTGTGT
156 R1	ACTACGTCTCT
156 R2	ACTACGTCTCT
239 R1	AGTACGAGAGT
239 R2	AGTACGAGAGT

Table S2. Percentages of replicate 1 (R1) and 2 (R2) of total sequences from classified taxonomic groups at each sampling time point that could not be further classified at the class, order, family, genera levels (<50% cutoff of RDP Classifier).

**Bacterial phyla unclassified at class level**	**105 days**		**142 days**		**156 days**		**239 days**	
	**R1**	**R2**	**R1**	**R2**	**R1**	**R2**	**R1**	**R2**
Proteobacteria	0.3121	0.2469	1.1930	1.0437	1.2407	0.7239	0.5307	0.6094
Chloroflexi	0.3277	0.1710	0.5086	0.5510	0.4913	0.4607	0.1628	0.3522
Firmicutes	0.2575	0.1456	0.1665	0.1748	0.0833	0.0376	0.0425	0.1901
Bacteroidetes	0.2107	0.2026	0.0555	0.1960	0.0874	0.1504	0.0212	0.0783
Verrucomicrobia	0.0390	0.0570	0.0000	0.0318	0.0125	0.0188	0.0000	0.0335

**Bacterial classes unclassified at order level**	**105 days**		**142 days**		**156 days**		**239 days**	
	**R1**	**R2**	**R1**	**R2**	**R1**	**R2**	**R1**	**R2**
Deltaproteobacteria	2.9728	1.9946	2.8022	1.6953	4.2510	1.2739	2.4202	2.0965
Alphaproteobacteria	3.1445	1.7413	1.8219	1.4781	1.1158	0.4137	0.8280	1.9064
Gammaproteobacteria	1.8258	1.2157	1.7756	1.4516	0.8993	0.3337	0.9412	2.1971
Clostridia	0.0312	0.1456	0.1387	0.1801	0.0250	0.0423	0.3468	0.5926
Spartobacteria	0.3121	0.0886	0.0555	0.1218	0.0416	0.0141	0.0283	0.0335
Betaproteobacteria	0.0312	0.0127	0.0185	0.0212	0.0333	0.0141	0.0283	0.0559
Actinobacteria	0.0000	0.0000	0.0092	0.0053	0.0125	0.0047	0.0000	0.0000
Acidobacteria Gp1	0.0000	0.0000	0.0092	0.0053	0.0000	0.0000	0.0000	0.0000

**Bacterial orders unclassified at family level**	**105 days**		**142 days**		**156 days**		**239 days**	
	**R1**	**R2**	**R1**	**R2**	**R1**	**R2**	**R1**	**R2**
Rhizobiales	0.8505	1.8363	3.5790	3.7084	4.7673	3.3421	2.7245	4.1874
Gammaproteobacteria incertae sedis	0.1717	0.3862	0.3977	0.4503	0.4372	0.4137	0.4883	0.6988
Rhodospirillales	0.2185	0.0760	0.3977	0.2755	0.1457	0.1551	0.1698	0.2180
Chromatiales	0.0156	0.0000	0.1202	0.1113	0.0416	0.0423	0.0354	0.4976
Burkholderiales	0.1561	0.1963	0.0555	0.0212	0.0458	0.0141	0.0495	0.2069
Bacillales	0.0078	0.0380	0.0462	0.1324	0.0541	0.1034	0.0071	0.0391

Chlamydiales	0.0156	0.0760	0.0277	0.1113	0.0291	0.0423	0.0283	0.0391
Clostridiales	0.0390	0.0380	0.0092	0.0636	0.0000	0.0047	0.0354	0.0671
Myxococcales	0.0000	0.0000	0.0277	0.0159	0.1457	0.0423	0.0000	0.0000
Sphingobacteriales	0.0234	0.0633	0.0092	0.0212	0.0000	0.0282	0.0000	0.0000
Actinomycetales	0.0078	0.0127	0.0000	0.0000	0.0000	0.0235	0.0000	0.0224
Bacteroidales	0.0156	0.0063	0.0092	0.0000	0.0000	0.0000	0.0000	0.0112
Lactobacillales	0.0078	0.0000	0.0092	0.0053	0.0042	0.0047	0.0000	0.0056
Alphaproteobacteria incertae sedis	0.0000	0.0000	0.0000	0.0106	0.0167	0.0000	0.0000	0.0000
Solirubrobacterales	0.0000	0.0000	0.0000	0.0106	0.0000	0.0000	0.0000	0.0112
Flavobacteriales	0.0000	0.0000	0.0000	0.0000	0.0000	0.0000	0.0000	0.0056
Caulobacterales	0.0000	0.0000	0.0000	0.0053	0.0000	0.0000	0.0000	0.0000
Nitrosomonadales	0.0000	0.0000	0.0000	0.0053	0.0000	0.0000	0.0000	0.0000

**Bacterial families unclassified at genus level**	**105 days**		**142 days**		**156 days**		**239 days**	
	**R1**	**R2**	**R1**	**R2**	**R1**	**R2**	**R1**	**R2**
Pseudomonadaceae	4.9860	4.7173	7.1581	4.5031	3.7514	3.0037	1.6206	4.2489
Sphingomonadaceae	6.0627	19.3567	0.9988	1.0066	0.3830	0.6722	0.0566	0.2516
Anaerolineaceae	2.4423	0.7155	3.4126	2.7760	3.0269	1.8332	0.2972	0.5199
Planctomycetaceae	0.5930	0.5066	1.2670	1.9443	1.7737	2.2516	0.4741	1.0622
Rhodospirillaceae	0.7335	0.9814	2.1178	2.3257	1.1366	0.8790	0.5520	0.7939
Bradyrhizobiaceae	0.3199	0.5762	1.0728	1.2344	1.8236	0.7051	0.6511	1.1629
Beijerinckiaceae	0.3979	0.4116	0.7861	0.9801	0.3622	0.2632	0.3114	0.6038
Erythrobacteraceae	0.3589	0.3926	0.3699	0.4503	0.5496	0.5923	0.3963	0.7995
Xanthobacteraceae	0.1092	0.2659	0.4532	0.5986	0.4663	0.4278	0.3821	0.5311
Acetobacteraceae	0.1326	0.1520	0.5549	0.7788	0.2706	0.3055	0.3680	0.4305
Rhodocyclaceae	0.0702	0.0570	0.2312	0.3020	0.3039	0.2209	0.1981	0.4417
Opitutaceae	0.3277	0.2026	0.3514	0.1748	0.1041	0.0564	0.2265	0.3466
Chitinophagaceae	0.1873	0.3356	0.1480	0.2066	0.1457	0.2867	0.0354	0.1677
Rhodobacteraceae	0.3355	0.2216	0.2220	0.2437	0.1207	0.1504	0.0212	0.0280
Clostridiaceae 4	0.0858	0.1393	0.0370	0.0848	0.0333	0.0047	0.0283	0.0224
Caulobacteraceae	0.0468	0.0886	0.0555	0.0689	0.0125	0.0188	0.0000	0.0112
Hyphomicrobiaceae	0.0546	0.0063	0.0740	0.0053	0.0583	0.0094	0.0495	0.0056

Veillonellaceae	0.0312	0.0317	0.0277	0.0053	0.0208	0.0235	0.0283	0.0559
Phyllobacteriaceae	0.0078	0.0380	0.0185	0.0159	0.0208	0.0282	0.0283	0.0391
Paenibacillaceae 1	0.0156	0.0063	0.0370	0.0265	0.0167	0.0141	0.0000	0.0447
Streptosporangiaceae	0.0000	0.0063	0.0092	0.0000	0.0000	0.0047	0.0000	0.1398
Porphyromonadaceae	0.0546	0.0633	0.0092	0.0000	0.0000	0.0094	0.0000	0.0168
Xanthomonadaceae	0.0078	0.0253	0.0277	0.0000	0.0208	0.0000	0.0071	0.0224
Thermomonosporaceae	0.0000	0.0127	0.0092	0.0053	0.0042	0.0094	0.0000	0.0503
Rhizobiales incertae sedis	0.0156	0.0317	0.0000	0.0053	0.0083	0.0141	0.0071	0.0056
Ruminococcaceae	0.0156	0.0063	0.0000	0.0053	0.0042	0.0282	0.0000	0.0224
Hyphomonadaceae	0.0156	0.0000	0.0000	0.0000	0.0000	0.0094	0.0000	0.0391
Cytophagaceae	0.0078	0.0000	0.0185	0.0212	0.0000	0.0141	0.0000	0.0000
Clostridiaceae 2	0.0390	0.0127	0.0000	0.0000	0.0000	0.0000	0.0071	0.0000
Erysipelotrichaceae	0.0312	0.0253	0.0000	0.0000	0.0000	0.0000	0.0000	0.0000
Sphingobacteriaceae	0.0156	0.0190	0.0000	0.0000	0.0000	0.0094	0.0000	0.0112
Lachnospiraceae	0.0000	0.0000	0.0185	0.0053	0.0000	0.0094	0.0000	0.0112
Polyangiaceae	0.0000	0.0000	0.0000	0.0000	0.0291	0.0141	0.0000	0.0000
Cystobacteraceae	0.0000	0.0127	0.0092	0.0000	0.0042	0.0000	0.0071	0.0056
Oxalobacteraceae	0.0312	0.0000	0.0000	0.0000	0.0000	0.0000	0.0071	0.0000
Peptococcaceae 2	0.0000	0.0000	0.0185	0.0000	0.0000	0.0000	0.0142	0.0056
Flavobacteriaceae	0.0156	0.0000	0.0000	0.0053	0.0000	0.0000	0.0000	0.0168
Rhodobiaceae	0.0000	0.0000	0.0000	0.0000	0.0208	0.0000	0.0071	0.0056
Alcaligenaceae	0.0000	0.0000	0.0000	0.0053	0.0125	0.0094	0.0000	0.0000
Comamonadaceae	0.0078	0.0000	0.0000	0.0000	0.0000	0.0094	0.0000	0.0000
Methylocystaceae	0.0000	0.0000	0.0000	0.0000	0.0000	0.0000	0.0000	0.0112
Thermoanaerobacteraceae	0.0000	0.0000	0.0000	0.0106	0.0000	0.0000	0.0000	0.0000
Clostridiaceae 1	0.0000	0.0000	0.0000	0.0000	0.0000	0.0000	0.0000	0.0056
Neisseriaceae	0.0000	0.0000	0.0000	0.0000	0.0000	0.0000	0.0000	0.0056
Parachlamydiaceae	0.0000	0.0000	0.0000	0.0053	0.0000	0.0000	0.0000	0.0000
Enterobacteriaceae	0.0000	0.0000	0.0000	0.0000	0.0042	0.0000	0.0000	0.0000

Table S3. Significant correlations (*P* < 0.05) between biofilter operational/functional parameters and the percentages of total sequences from the top 40 most abundant detected genera.

Biofilter functional/operational parameter	Genera	Spearman´s Rank Correlation Coefficient (ρ)	Significance (*P* value)
**EBRT**	*Xanthobacter*	-0.772	0.025
**Elimination Capacity**	*Asticcacaulis*	-0.781	0.022
	Subdivision3 genera incertae sedis	-0.732	0.039
**Evenness (E)**	*Sphaerobacter*	0.762	0.028
	Haloferula	0.881	0.004
	Sphingomonas	0.81	0.015
	Mesorhizobium	0.81	0.015
	Rhodoplanes	0.786	0.021
	Brucella	0.786	0.021
**Evenness (J)**	*Sphaerobacter*	0.929	0.001
	Haloferula	0.905	0.002
	Sphingomonas	0.857	0.007
	Rhodoplanes	0.714	0.047
	Brucella	0.81	0.015
**Inverse Simpson index**	*Gp3*	0.714	0.047
	Sphaerobacter	0.905	0.002
	Haloferula	0.952	<0.001

	Xanthobacter	0.714	0.047
	Sphingomonas	0.929	0.001
	Brucella	0.786	0.021
**Moisture content**	*Rhodobacter*	0.732	0.039
	Pasteuria	0.878	0.004
	Opitutus	0.927	0.001
	Pseudonocardia	-0.781	0.022
**Observed OTUs**	*Sphaerobacter*	0.881	0.004
	Haloferula	0.881	0.004
	Xanthobacter	0.738	0.037
	Sphingomonas	0.976	<0.001
	Rhodoplanes	0.714	0.047
**pH**	*Gemmatimonas*	-0.878	0.004
	Ohtaekwangia	0.781	0.022
	Rhodoplanes	-0.781	0.022
**Removal efficiency**	*Brevundimonas*	-0.732	0.039
	Asticcacaulis	0.781	0.022
	Hydrogenophaga	-0.732	0.039
	Achromobacter	-0.781	0.022
	Gemmatimonas	0.878	0.004
	Subdivision3 genera incertae sedis	0.732	0.039
	Steroidobacter	0.878	0.004
	Gp6	0.878	0.004
	Stenotrophomonas	-0.83	0.011
	Kaistia	0.878	0.004

	Ohtaekwangia	-0.976	<0.001
	Truepera	0.781	0.022
	Alkaliphilus	-0.781	0.022
**Shannon index**	*Sphaerobacter*	0.976	<0.001
	Haloferula	0.929	0.001
	Sphingomonas	0.929	0.001
	Brucella	0.786	0.021
**Styrene inlet concentration**	*Asticcacaulis*	-0.976	<0.001
	Subdivision3 genera incertae sedis	-0.878	0.004
	Gp6	-0.878	0.004
	Stenotrophomonas	0.781	0.022
	Ohtaekwangia	0.781	0.022
**Styrene inlet load**	*Asticcacaulis*	-0.781	0.022
	Subdivision3 genera incertae sedis	-0.732	0.039
**Styrene outlet concentration**	*Brevundimonas*	0.732	0.039
	Asticcacaulis	-0.781	0.022
	Hydrogenophaga	0.732	0.039
	Achromobacter	0.781	0.022
	Gemmatimonas	-0.878	0.004
	Subdivision3 genera incertae sedis	-0.732	0.039
	Steroidobacter	-0.878	0.004

Gp6	-0.878	0.004
Stenotrophomonas	0.83	0.011
Sorangium	0.732	0.039
Kaistia	-0.878	0.004
Ohtaekwangia	0.976	<0.001
Truepera	-0.781	0.022
Alkaliphilus	0.781	0.022

Figure S1. Rarefaction curves at 3% cutoff for sampling days 105, 142, 156, and 239. Error bars represent 95% confidence intervals.

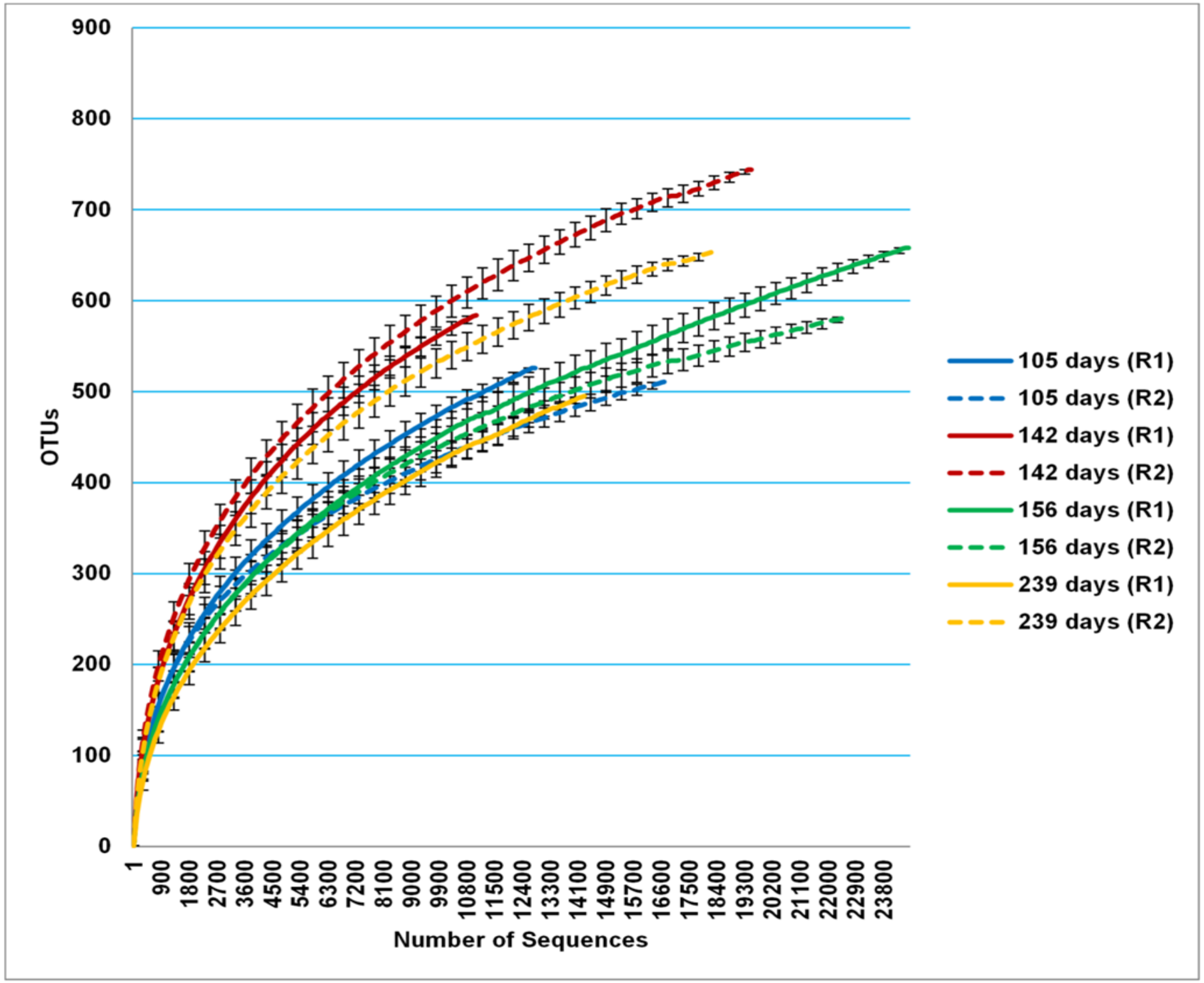

Figure S2. Non-metric multidimensional scaling (NMDS) plot of replicate samples 1 and 2 (R1 and R2, respectively) from sampling days 105, 142, 156 and 239.

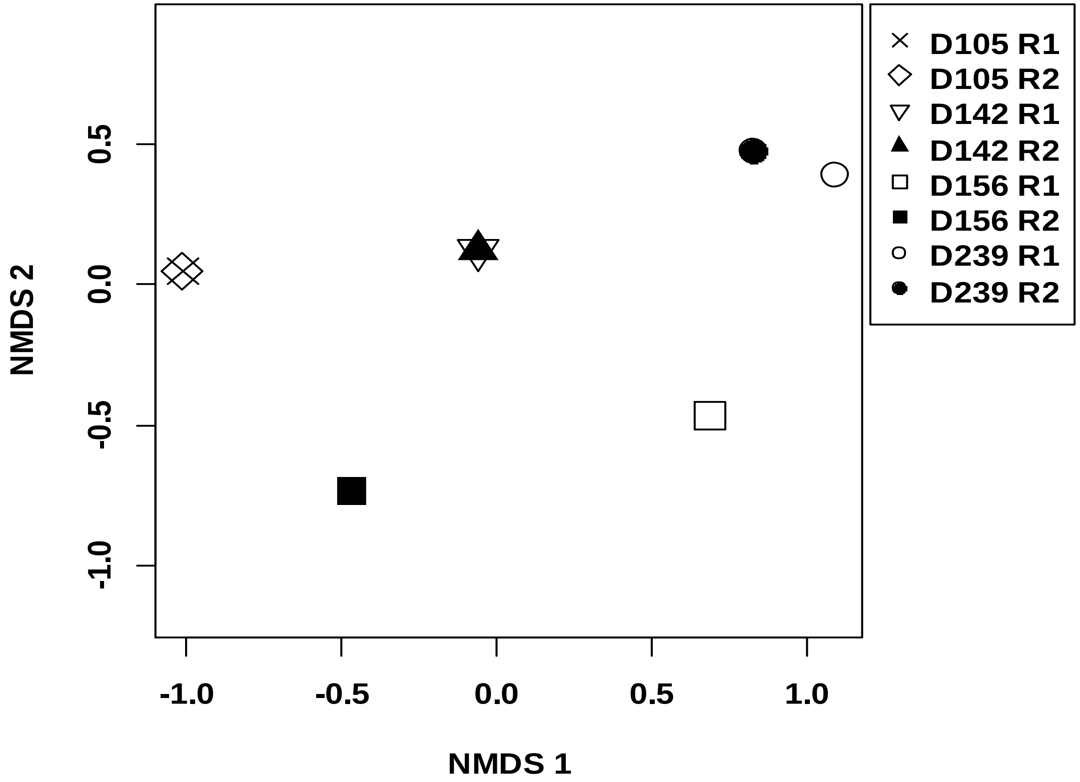

Figure S3 Percentage of total bacterial sequences (mean of replicates) from the top 40 detected genera in the 4 sampling time points.
Error bars represent the lower and upper values of actual replicates. Sequences labeled as Unclassified (Genera) could not be assigned to any particular genera using the Ribosomal Database Project Classifier at the selected bootstrap confidence estimate threshold of 50%.
Subdivision 3 incertae sedis belongs to the phylum Verrucomicrobia.

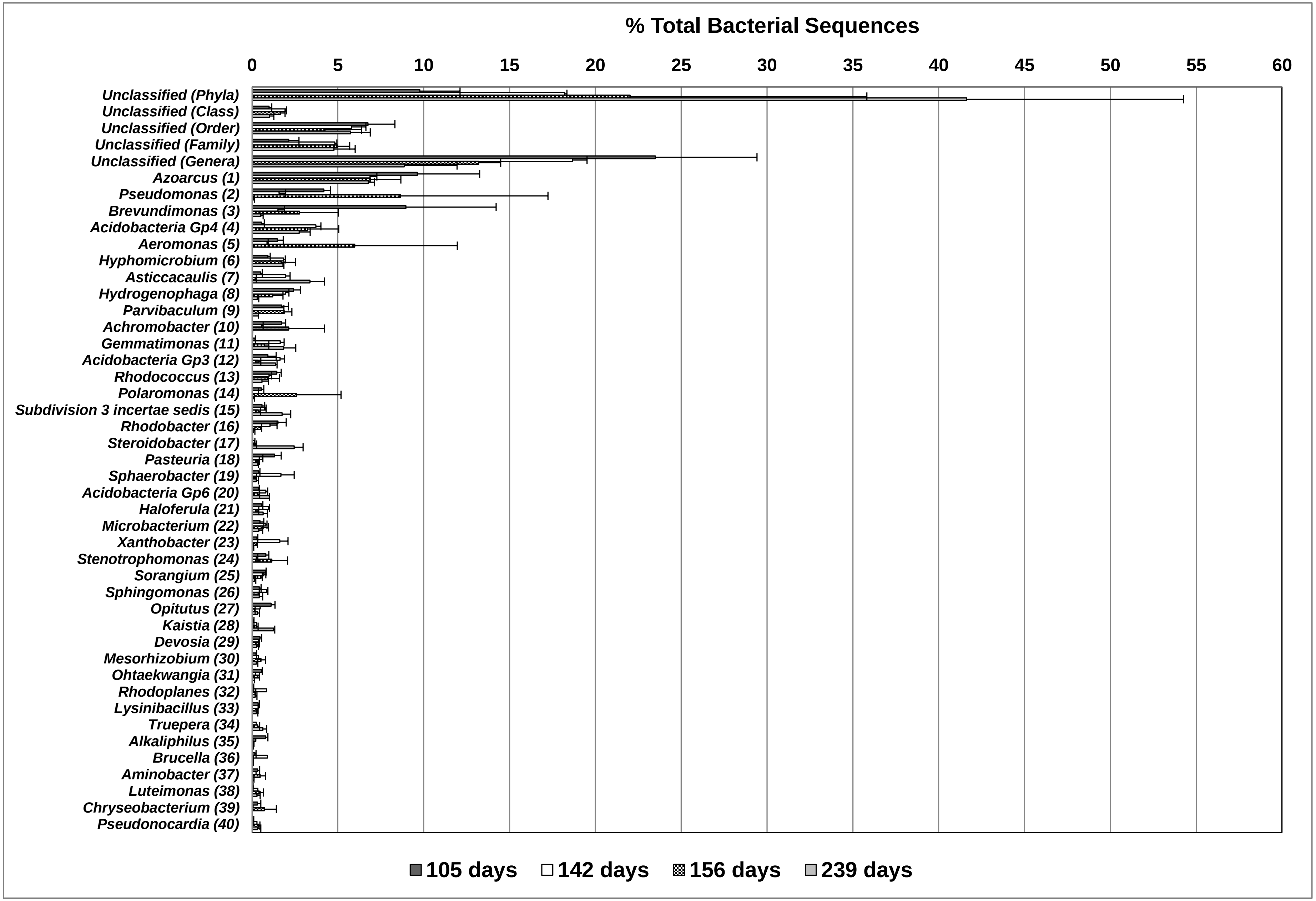
% Total Bacterial Sequences
0
5
10
15
20
25
30
35
40
45
50
55
60
Unclassified (Phyla)
Unclassified (Class)
Unclassified (Order)
Unclassified (Family)
Unclassified (Genera)
Azoarcus (1)
Pseudomonas (2)
Brevundimonas (3)
Acidobacteria Gp4 (4)
Aeromonas (5)
Hyphomicrobium (6)
Asticcacaulis (7)
Hydrogenophaga (8)
Parvibaculum (9)
Achromobacter (10)
Gemmatimonas (11)
Acidobacteria Gp3 (12)
Rhodococcus (13)
Polaromonas (14)
Subdivision 3 incertae sedis (15)
Rhodobacter (16)
Steroidobacter (17)
Pasteuria (18)
Sphaerobacter (19)
Acidobacteria Gp6 (20)
Haloferula (21)
Microbacterium (22)
Xanthobacter (23)
Stenotrophomonas (24)
Sorangium (25)
Sphingomonas (26)
Opitutus (27)
Kaistia (28)
Devosia (29)
Mesorhizobium (30)
Ohtaekwangia (31)
Rhodoplanes (32)
Lysinibacillus (33)
Truepera (34)
Alkaliphilus (35)
Brucella (36)
Aminobacter (37)
Luteimonas (38)
Chryseobacterium (39)
Pseudonocardia (40)
105 days
142 days
156 days
239 days
